# Frequently repeated measurements -our experience of collecting data with SMS

**DOI:** 10.1186/s12874-020-01013-y

**Published:** 2020-05-19

**Authors:** I. Axén, I. Jensen, E. Butler Forslund, B. Grahn, V. Jørgensen, C. H. Opava, L. Bodin

**Affiliations:** 1 Institute of Environmental Medicine, Unit of Intervention and Implementation Research for Worker Health, Karolinska Institutet, Nobels väg 13, S- 171 77 Stockholm, Sweden; 2 Rehab Station Stockholm, Research and Development Unit, Stockholm, Sweden; 3 Department of Neurobiology, Care Sciences and Society, Division of Physiotherapy, Karolinska Institutet, Huddinge, Sweden; 4 Department of Clinical Sciences Lund, Orthopedics, Faculty of medicine, Lund University, Lund, Sweden; 5 Department of Research and Development, Region Kronoberg, Växjö, Sweden; 6 Research Departement, Sunnaas Rehabilitation Hospital, Bjørnemyrveien 11, N-1453 Bjørnemyr, Norway

**Keywords:** Ecological momentary assessment, Repeated measures, Text message, Compliance

## Abstract

**Background:**

As technology is advancing, so are the possibilities for new data collection methods in research, potentially improving data quality and validity of the results. In Sweden, a system using frequent repeated data collection using text messages, SMS Track, has been used in clinical research for more than a decade. In this paper, compliance with repeated text message questions was examined across five different studies, i.e. if compliance was

1: associated with study-specific factors (age or gender of the subjects, the condition, its’ severity or course, i.e. improvement, relapse or steady state) and/or.

2: associated with the methodology itself (the question being asked, the frequency and number of questions, duration of data collection, initial compliance or the management of the system).

**Methods:**

Descriptive comparisons were done across five studies. Three studies were collecting weekly responses over at least 52 weeks (“Weekly studies”) and were used to investigate the effect of age, sex and pain severity on compliance, the effect of early compliance for late compliance, and finally the early occurrence of two successive weeks with non-compliance.

**Result:**

Compliance was excellent across all five studies, and only influenced somewhat by age, sex and pain-level. The factor “study” remained significant in the final model thus the observed differences may be a result of the conditions studied but does not seem to be attributable to severity or development of these conditions. Number and frequency of questions did not influence compliance, nor did study duration.

**Conclusions:**

Compliance was excellent in the included studies and was not affected by population factors. However, differences in compliance were observed that cannot be easily explained and warrant further investigation. In particular, the nature of the variables or the management of the study are potential areas for further investigations.

## Background

Data in prospective studies have traditionally been collected using interviews and questionnaires, and have often been restricted to before/after measurements, i.e. how the patient was doing at baseline (before the intervention) compared to some time later (after the intervention or the passing of time), often at 3, 6 or 12 months. Previously, the administrative burden of collecting data with these methods, as well as the challenge of get responders to comply, made frequent data collection unfeasible. A recent study concluded that this method of selecting rather arbitrary time points may render the results unreliable [1]. This is certainly the case in fluctuating conditions, such as many musculoskeletal conditions and chronic illnesses, where the selected measuring point may be reflecting a random point in the individual trajectory, and not be a true measure of improvement or decline of the condition.

Recent technological advances have permitted frequently repeated measures, using mobile phones and the internet. As people worldwide are becoming more IT literate, these methods are appealing in terms of availability, user-friendliness, costs and ultimately, data quality.

In Western societies, IT penetration is close to 100%. In Sweden, nearly 100% of the population own their own mobile phone and have access to and use the internet daily [2]. The low and middle income countries are not far behind [3].

A system whereby a participant receives automated text messages, SMS, at frequent intervals, and reports their status in a reply SMS requires a minimal effort from the respondent. People carry the phone with them at all times, and the tendency is that of being constantly on-line. This means that questions via SMS can be answered in real time, with a minimum of recall bias, and in real life circumstances, known as ecological momentary assessment [4]. This term describes a new way of collecting data, reflecting the fact that the respondent may actually answer in their own environment (ecological) when the incident occurs (momentary), not waiting for an appointment in a lab after the incident of interest has passed. Research using this type of frequent assessment generally show excellent response-rates [5–7].

A system called SMS-Track®, specifically developed for research, has been used for about 10 years in the Scandinavian countries [8]. It has been scrutinized for user-friendliness and was found to have high compliance [9]. Subjects have been followed with up to three weekly questions for up to 2 years with a compliance of over 90% [10, 11]. Compared to using paper questionnaires, the system also carries low costs [12]. The system uses a web-based interface, whereby the data are stored instantly in an on-line file, accessible to the researcher and easily downloadable for analysis. As such, errors related to typing in and transfer of data for analysis are minimized.

In all data collection, scrutiny is warranted before relying on the data and hence, on the conclusions drawn from the study. In the case of collecting data with SMS, compliance, i.e. the response rate, is of the utmost importance if data imputation should be kept to a minimum. Specifically: it is important to assess if compliance is dependent on subject-related factors or on the method itself. Concerning text messages as a data collection tool; if there are certain individuals (who for some reason cannot manage the technology) or types of questions where this technology is not suitable. In addition, it will be important to know if measuring frequency, time of measurement (i.e. during holidays) or number of questions influence the compliance, or if the “severity” of the condition does, e.g. if individuals who are severely affected by a condition comply differently than individuals who are less affected regarding condition-specific outcomes. Finally, it will be a concern if subjects comply differently when recovering from their condition compared with experiencing a “steady state”.

Data from five studies using the SMS Track system allowed these questions to be explored. Working with the system over the years, one hypothesis was that early compliance is “key” to long-term compliance.

Specifically, this paper aimed to investigate if compliance with frequent SMS questions was

1. associated with study-specific factors (age or gender of the subjects, the condition, its’ severity or course, i.e. improvement, relapse or steady state) and/or
2. associated with the methodology itself (the question being asked, the frequency and number of questions, duration of data collection, initial compliance or the management of the system).

## Method

### Data and settings

This paper utilized data from 5 prospective studies, all conducted in Sweden from 2011 through 2017 and all using SMS-Track as a data collection tool. The investigators in each of the included studies were trained by the same coordinator regarding response management and reminding, as previously tested and described [9]. In short, to optimize compliance, the weekly responses were closely monitored, and respondents who failed to answer 2 weeks in a row were called to make sure they understood that every response was important. The studies differed in terms of aims, populations, outcomes (and thus type and number of questions asked) and frequency of measure. All of the studies also collected data by other means besides using SMS, through interviews, clinical tests, patient records, questionnaires and registers. An overview of their characteristics is seen in Table 1.

1. The Maintenance Care “MC” Chiro study (principal Investigator (PI): Axén) [13]. This was a randomized clinical trial investigating 321 patients consulting a chiropractor in Sweden with low back pain (LBP). The trial aimed to investigate the effect of a preventive approach employed by chiropractors directed towards recurrent and persistent LBP and sent a weekly SMS for 52 weeks asking about the number of days with bothersome LBP: “*On how many days during the past week were you bothered by your LBP (i*.*e*. * affected in your daily activities or routines)*?”
2. The Spinal Cord Injury Prevention “SCIP” Falls study (PI: Skavberg Roaldsen) [14, 15]. This was an observational study of 224 individuals with Spinal Cord Injury (SCI) in Norway and Sweden aiming at investigating recurrent falls both in ambulant persons and wheelchair users. The subjects received a SMS every other week for 1 year asking if they had experienced any falls: *“Have you fallen the previous two weeks? (yes or no)”.* If the response was “yes”, the research team would call the individual for details in order to explore the incident further.
3. The Work-Up study (PI: Grahn) [16]. This was a cluster randomized controlled trial investigating a working population of 325 individuals with neck- and back-pain, who consulted in primary care. The trial aimed to investigate the effect of an improved dialogue between the patient, the employer and the physiotherapist to adapt work conditions to prevent sick listing and improve work ability. Every week for 1 year, the subjects received 3 SMS with questions concerning sick-leave, work ability and disability; *1) “Last week, how many days were you on sick leave?” 2) Last week, to what extent did your neck/back problems impair your work performance?”* and *3) Last week, to what degree did your neck/back problems hinder you in carrying out daily routines in family life and leisure?”*
4. The Physical Activity program for people with Rheumatoid Arthritis, “PARA” study (PI: Opava) [17]. This was an observational study investigating 220 patients with rheumatoid arthritis (RA) involved in a program to facilitate health-enhancing physical activity to improve health. The aim was to investigate the adherence to a physical activity program that included support by coaches. Two weekly SMS were sent for 2 years (104 weeks) asking about the number of circuit training sessions and additional days with free-living physical activity*: 1) “How many circuit-training sessions did you do the past week”?* and *2) “Besides the circuit training, how many additional days of the past week did you perform at least moderate-intensity physical activity for at least 30 minutes?”*
5. The Stress Prevention At work “SPA” study (PI: Jensen) [18]. This was a cluster randomized controlled trial among 121 individuals working in primary health care where an intervention to prevent work-related stress was carried out. The aim was to investigate the effectiveness of this intervention. Data were recorded for two periods, SPA I (the first 13 weeks of the intervention) and SPA II (starting at months 6 after the intervention and running for 26 weeks). In both periods, the participants received a weekly SMS asking about their level of stress*: “Stress means a state in which a person feels tense, restless, nervous or anxious or is unable to sleep at night because his/her mind is troubled all the time. Do you feel this kind of stress these days?”.*

**Table 1:** The number of subjects, inclusion and exclusion criteria and the baseline characteristics of the included subjects

Study N	Inclusion criteria	Exclusion criteria	Female %	Age, mean	Education/Work %	Health, 5 points %	Health EQ-5D mean	Pain NRS/VAS mean
**MC Chiro** 321	Non-specific LBP	Specific LBP	56.4	43	Heavy 10.9	Excellent 5.7	0.6925	5.27 (*n* = 300)
	Working age				Standing 31.8			
	Fluent in Swedish					Very Good 38.8		
					Sitting 46.1			
						Good 42.8		
						Somewhat 14.7		
						Poor 3.7		
**SCIP Falls** 224	Traumatic SCI	Complete injuries above C5-level,	22.9	49	< 9 years 31.5		0.4136	0.77
	> 18 years old				High school 30,6			
	Fluent in Swedish /Norwegian	Injuries below L5 level,						
		Injuries classified as AIS E^a^			University 37,9			
**Work-Up** 352	Early back and neck pain	Identified abuse, retirement pension, ongoing acute medical treatment, pregnancy	65.3	44	< 9 years 8.5	Excellent 7.1	0.5046	5.20 (*n* = 332)
	18–67 years				High school 50.0			
	Last year: working ≥4 weeks, short sick leave ≤60 days, Linton short version ≥40 p				University 21.9	Very Good 44.6		
					Other 19.3			
						Good 36.9		
						Somewhat 10.2		
						Poor 0.6		
**PARA** 220	RA	Already obtain physical activity levels aimed at in the study	81.4	59	< 9 years 18.2		EQ-5D thermometer 30.58	2.9
	18–75 years old				High school 20.9			
	Independent in daily living							
	Fluent in Swedish				University 50.5			
					Other 10.5			
**SPA** 106	Working age		70.5	46	High school 14.4	Very Good 25.7		
	Employed in primary care				University 66.7			
						Good 36.2		
						Somewhat 16.2		
						Poor 2.9		

*EQ-5D* Euro Qol 5 dimensions, *LBP* Low back pain, *SCI* Spinal Cord Injury, *AIS E*^a^ refers to normal sensibility and motor function, *RA* Rheumatoid Arthritis

### Ethical permission

All the studies had individual ethical permission from their local ethic committees; MC Chiro: 2007/1458–31/4, SCIP Falls: 2012/830–31/2, 2013/391–32, 2014/364–32, Work-Up: 2012/497, 2012/648, 2012/833, PARA: 2010/1232–31/1 and SPA: 2012/2200–31/5.

In addition, ethical approval for this specific merge and analysis of data was granted by the Swedish Ethical Review Authority (2017/961–31/5) for the Swedish data and by the Regional Committees for Medical and Health Research Ethics in Norway (2012/531) for the Norwegian data.

### Data analysis

The data for the five studies were rearranged to get a similar structure with respect to compliance over the available weeks. Three studies (MC Chiro, Work-Up and PARA) covered a time span of (at least) 52 weeks with weekly SMS replies. Due to this similarity, data from these three studies, hereafter called the “Weekly studies” were used for comprehensive analysis. The remaining two studies did not cover 52 weeks (SPA) or weekly messaging (SCIP Falls) and they were therefore included only in the descriptive comparisons.

Pain was investigated at baseline in four studies, all except SPA. In MC Chiro, the Numeric Rating Scale with categories 0–10 were used, and in SCIP Falls, Work-Up and PARA, the Visual Analog Scale (VAS) was used, where the respondent is asked to rate their pain on a horizontal line from 0 to 100. In our analysis, the VAS-score was treated like a categorical variable, in order to aggregate data from the Weekly studies [19]. Further, the pain variable was categorized into “Painclass” with mild pain 0–5, moderate pain 6–7 and severe pain 8–10 [20] in order to explore “severity”.

Time was represented with two different settings. The first setting, used in most of the descriptions and analysis in this paper, is the study week. This refers to the consecutive weeks the subjects obtained a SMS. For subjects in the Weekly studies, this number spans from 1 to 52. The second time setting in this paper, used to study compliance in relation to holidays, was the calendar week that corresponded to the subject’s study week.

The dependent variable in this paper was “compliance”, i.e. if the subjects answered the SMS question. The variable was therefore dichotomous, 0 = no answer and 1 = answer.

For descriptive purposes, compliance was presented in figures as percentage of missing answers; for all studies for 52 study weeks and in relation to calendar week. One study (PARA) collected data for 2 years, but for comparative purposes, the responses for the second year were omitted. Then, using data from the Weekly studies, figures of compliance stratified for sex, age and for pain severity categories were presented in this paper. A lowess method calculated a smoothed curve over the analyzed time period, in this case the 52 weeks. Lowess is described by Cleveland [21] as a robust locally weighted regression, and we applied it with a bandwidth of 0.6, which is a bit smaller than the default of the method (0.8). With this smaller value we found a less extreme smoothing which allowed us to show the existence of ups and downs in compliance over the time period.

Early and late compliance in this paper was defined as the initial and last 8 weeks of the study, respectively. A Poisson regression with the outcome “number of missing SMS during 8-week periods” at the start and at the end of the 52 weeks study schedule were used for the Weekly studies. Thus, the outcome was a number in the interval 0 to 8 (i.e. the number of missing SMS). The outcome parameter was the Relative Risk (RR) for missing SMS, or non-compliance. The RR was shown with a 95% confidence interval (CI), and *p*-value for a test of RR = 1.0, that is, no effect on compliance. Factors that were assumed to effect compliance were “study” (with MC Chiro serving as the reference), age (with age below 50 years as the reference category), sex (with males as the reference category) and baseline pain in three categories (mild pain as reference). We also introduced a combined variable with age (below and above 50 years) crossed with sex, using the category males < 50 years as the reference category. The Poisson regression belongs to the family of generalized linear models with a distribution according to the Poisson distribution and a logarithmic link function.

Finally, a time-to-event analysis was performed for the Weekly studies, where the event in this paper was the first occurrence of two consecutive weeks without answering SMS, as this was the suggested point of making contact with the respondent to ensure that they had understood the importance of answering every week. The analysis was based on a Cox proportional hazards model with “study”, age, sex and Painclass as the included variables. Results were presented with a curve equivalent to a survival curve, where the equivalence to death was the event described above. The Cox model also gives estimates of the Hazard Ratio (HR). The proportional hazards assumption of the Cox model was tested using Schoenfield residuals.

The statistical softwares STATA (version 15) and SPSS (version 25) were used for all analyses. Statistical significance was set to *p* < 0.05.

## Results

Some of the baseline characteristics of the individuals in the included studies are presented in Table 1. More variables were collected in each individual study, but the ones presented here were similar across studies and serve as ground for comparison of the included subjects in this paper. All studies had data on age, sex and education/work, and all had a variable describing the health of the subjects. For the three Weekly studies, pain was also recorded at baseline.

There are clearly some differences in the study populations. Overall, most of the subjects were in their 40’s, but the subjects in PARA were older. In SCIP Falls, only a fifth were female, whereas in PARA, four fifths were. In Work-Up, a fifth had a university education, and in SPA, two thirds did. Concerning the health status of these individuals, the MC Chiro subjects were rating their health as good, compared to the poorest health that were found among the SCIP Falls and PARA participants. Comparing the Weekly studies, PARA is different from the other two concerning sex (more females), age (older), and pain (milder).

Overall compliance was slightly different between studies, see Table 2 for details. The most SMS-extensive study, Work-Up, received 49,607 replies to the 54,912 questions sent, an overall compliance of 90.3%.

**Table 2:** Overall SMS compliance stratified by study and question

Study	Question(s)	N of SMS according to study protocol	Overall Compliance n (%)
*Studies with 52 weeks “Weekly” studies*			
MC Chiro	Number of bothersome pain days	16,692	16,505 (98.9)
Work-Up	Number of days of sick leave	18,304	16,601 (90.7)
	Productivity loss	18,304	16,531 (90.3)
	Disability	18,304	16,475 (90.0)
PARA	Number of circuit training sessions	11,440	10,084 (88.1)
	Number of additional days with 30 min activity sessions	11,440	9959 (87.1)
*Studies with less than 52 weeks*			
SCIP Falls^a)^ (25 weeks)	Fallen or not (Yes/no)	5350	5175 (96.7)
SPA I (13 weeks)	Level of stress	1222	1192 (97.5)
SPA II (26 weeks)	Level of stress	2288	2209 (96.5)

^a^Every other week for 49 weeks

**Fig. 1:**
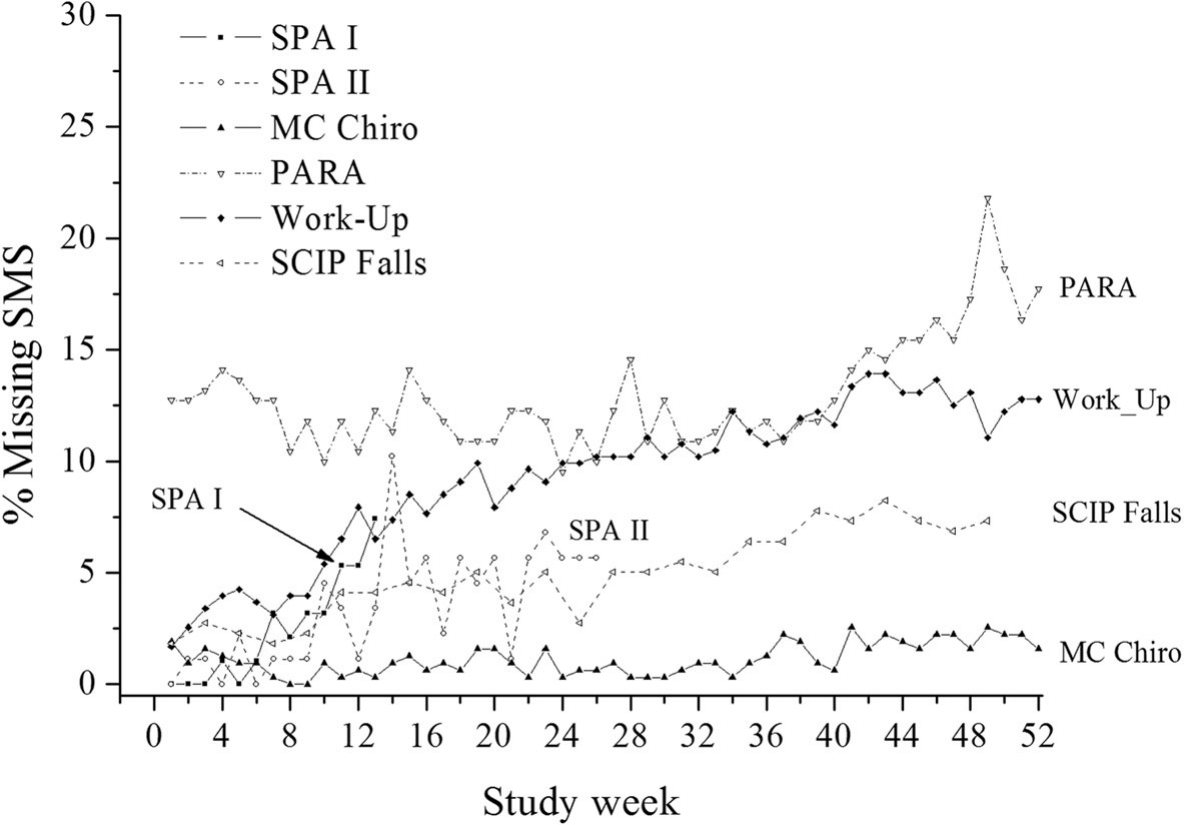
Non-compliance (percentage of missing answers) in each of the five studies over the first 52 weeks

Overall, the compliance in all five studies started excellently (few missing SMS), and gradually lost responses over time (Fig. 1, the second year removed from PARA). The exceptions were PARA with a lower initial compliance compared to the rest, and MC Chiro, that kept a high compliance throughout.

**Fig. 2:**
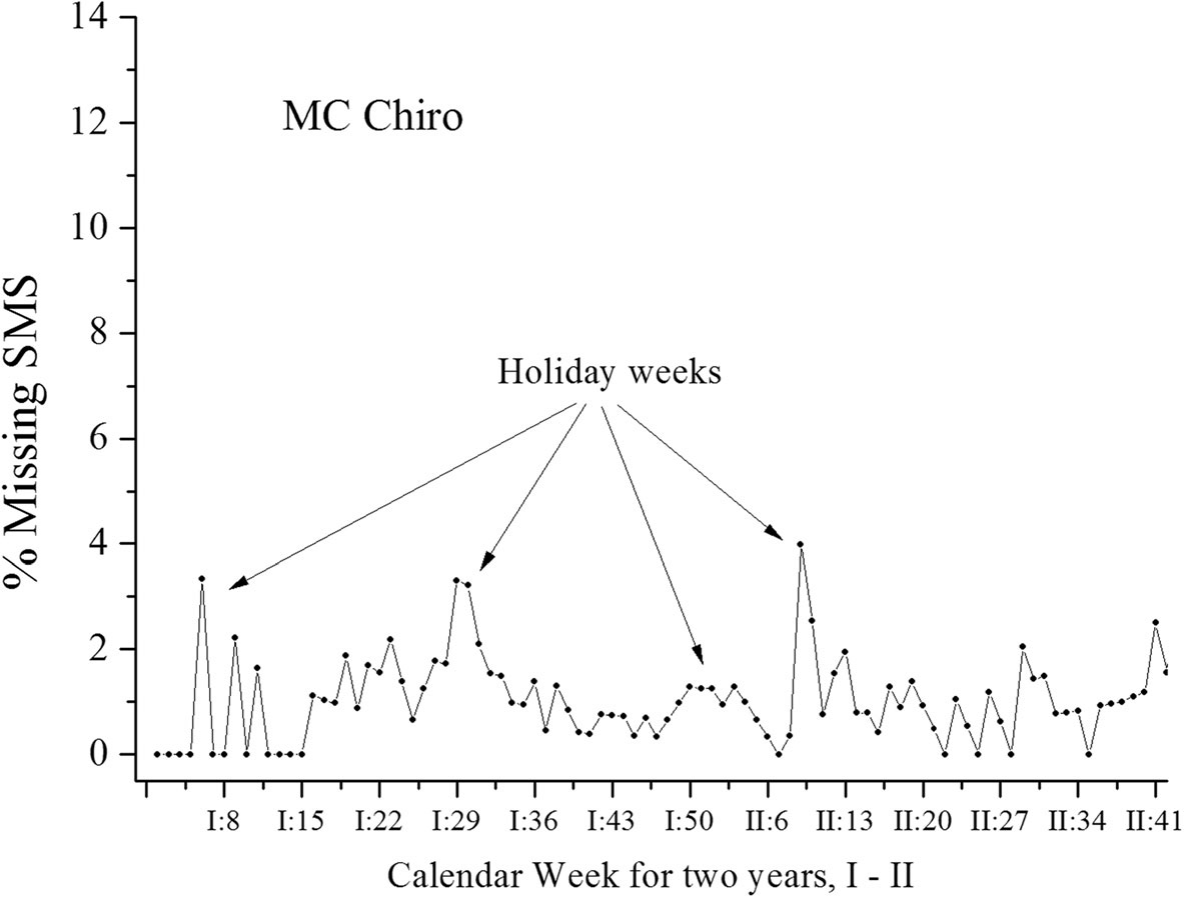
Non-compliance (percentage of missing answers) in MC Chiro over 52 weeks

During the Swedish holidays of Christmas and Easter, as well as summer, compliance was clearly lower (Fig. 2; the percentage of missing responses per calendar week for MC Chiro). All studies showed a similar tendency of lower compliance for holiday periods (data not shown).

In relation to age and sex of the subjects, compliance as percentage of missing answers is shown in Table 3 for the Weekly studies.

**Table 3:** Compliance in the Weekly studies stratified by age, sex and Painclass. The figures show the number of responses according to the number of SMS sent for each strata and study

	MC	Work-Up	PARA
Age & sex^a^			
-49 years, Male	3713/3744; 99.17%	3469/3744; 92.65%	113/156; 72.44%
-49 years, Female	6200/6292; 98.54%	7074/7904; 89.50%	1347/1404; 95.94%
50+ years, Male	1786/1820; 98.13%	2204/2444; 90.18%	1815/1976; 91.85%
50+ years, Female	3001/3016; 99.50%	3702/4056; 91.27%	6809/7904; 96.15%
Painclass^a^			
≤ 5	8002/8060; 99.28%	8336/9204; 90.57%	7906/8892; 88.91%
6–7	4942/5044; 97.98%	4817/5252; 91.71%	1479/1716; 86.19%
8–10	2474/2496; 99.12%	2533/2808; 90.21%	606/728; 83.24%

^a^The total numbers of SMS are lower here than in Table 2 due to some missing data on age, sex and pain

**Fig. 3:**
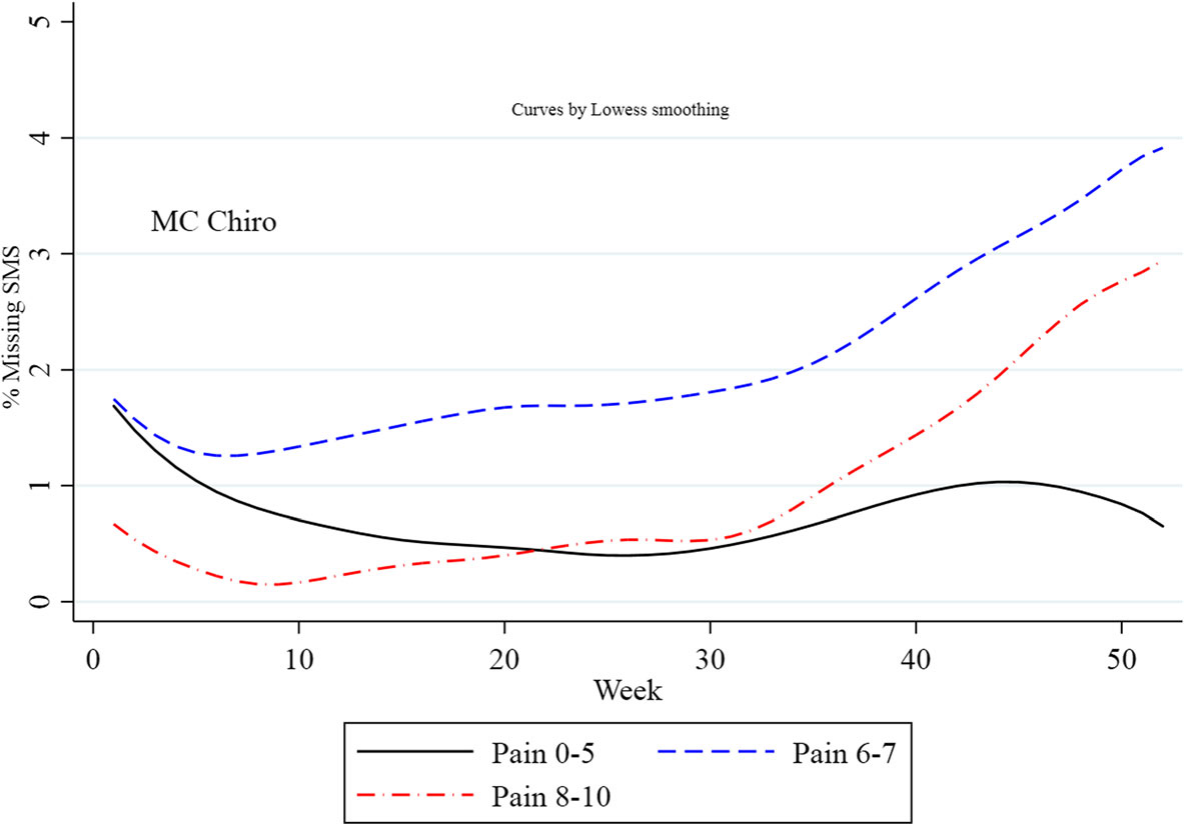
Non- compliance (percentage of missing answers) in relation to baseline pain intensity level; mild (0–5), moderate (6, 7) and severe (8–10), in MC Chiro over 52 weeks

Compliance in relation to baseline pain intensity: Mild (Pain levels 0–5), Moderate (Pain levels 6–7) and Severe (Pain levels 8–10), for the Weekly studies combined, is also presented in Table 3. Overall, pain severity does not seem to affect compliance. However, studying MC Chiro alone, Fig. 3, show that the respondents with moderate and severe pain have poorer compliance compared to the respondents reporting mild pain.

There was a tendency in PARA that those with severe pain had less compliance at the end of the study period but this observation was based on only 14 subjects and not found for the other studies.

The Poisson regression analysis for the Weekly studies, investigated first whether early and late compliance was affected by age, sex, Painclass and “study”. Further, if early compliance was predictive of late compliance, i.e. if answering the SMS the first 8 weeks was predictive of answering the last 8 weeks of the study. This model was also adjusted for “study”, age, sex and baseline pain intensity, as shown in Table 4.

**Table 4:** Analysis of number of missing SMS (0 thru 8) for the first 8 weeks and the last 8 weeks for the Weekly studies. A Poisson regression model was used and the Relative Risk (RR) is the outcome parameter, and RR = 1.0 indicates no effect, presented also with confidence intervals, and RR > 1.0 indicates higher risk for non-compliance

Study	MC Chiro	Work-Up	PARA
Variables in model			
Age > 50 (reference ≤50)	4.27 (1.73–10.50)	0.92 (0.59–1.42)	4.38 (1.94–9.91)
Sex (reference males)	0.20 (0.07–0.55)	0.59 (0.39–0.90)	1.07 (0.73–1.57)
Pain class 6–7 (reference < 6)	1.23 (0.52–2.89)	1.15 (0.70–1.89)	1.27 (0.87–1.88)
Pain class 8–10 (reference < 6)	0.39 (0.05–3.05)	1.62 (0.96–2.75)	1.48 (0.90–2.44)
Study (with all background variables)	1.0 (reference)	3.41 (2.13–5.44)	9.36 (5.81–15.09)
Age > 50 (reference ≤50)	0.52 (0.25–1.08)	1.17 (0.93–1.47)	1.23 (0.76–1.98)
Sex (reference males)	1.56 (0.82–2.97)	1.68 (1.31–2.16)	1.37 (0.97–1.93)
Pain class 6–7 (reference < 6)	5.05 (2.38–10.72)	0.86 (0.67–1.10)	0.94 (0.68–1.30)
Pain class 8–10 (reference < 6)	3.28 (1.30–8.28)	0.97 (0.72–1.31)	1.54 (1.07–2.22)
#Missing first 8 weeks	1.40 (0.95–2.07)	1.43 (1.37–1.50)	1.35 (1.31–1.39)
Study (with all background variables + #missing first 8 weeks)	1.0 (reference)	5.21 (3.83–7.11)	3.99 (2.82–5.65)

Older *age* seems to be associated with poorer compliance the first 8 weeks of the study (although not for Work-Up) but does not influence late compliance. Women seem to be good compliers initially but late compliance has no association with *sex*. *Pain intensity* at baseline did not significantly influence early or late compliance, except for MC Chiro, where low pain was associated with better compliance at both times. The most important factor affecting compliance, however, was “study”. Thus, the available baseline characteristics explained far less of the compliance than the study itself. The initial early compliance was found to be a predictor for late compliance.

**Fig. 4:**
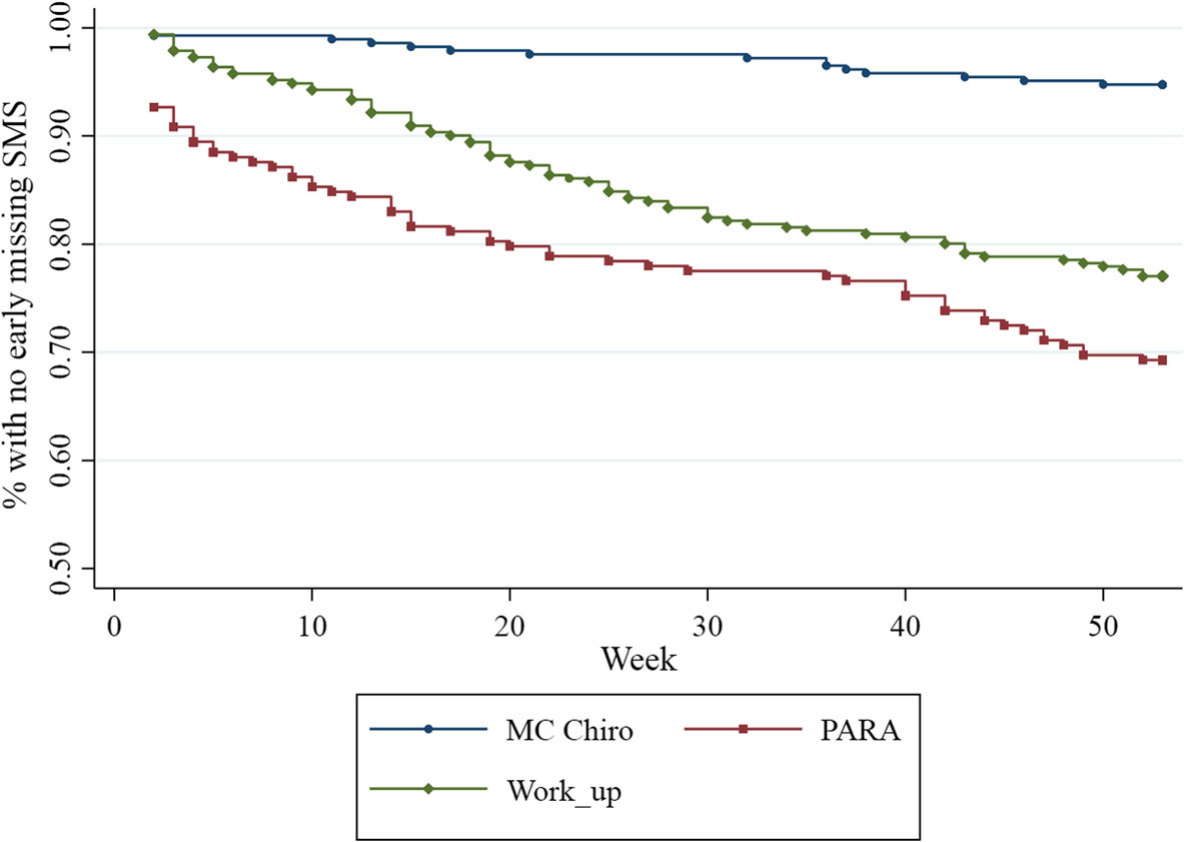
Curve showing occurrence of early missing SMS for the Weekly studies

The percentage of subjects in the Weekly studies that had no early missing SMS was plotted against the study week (Fig. 4). There was a substantial difference between the three Weekly studies, where MC Chiro had the best outcome with very few individuals with missing SMS.

The results of the Cox proportional hazard model are shown in Table 5. The hazard ratio (HR) was somewhat lower for higher age but not statistically significant. Sex and pain had none or small effects on early missing SMS, and, as with the other analyses, the statistically significant effect was found for “study”, with PARA displaying the highest value, HR 6.17 (95% CI 3.38–11.28, *p* < 0.001).

**Table 5:** Analysis of time, in weeks, from study start to the first occurrence of two consecutive weeks with missing SMS for the Weekly studies. A Cox proportional hazard model was used and the outcome parameter was the Hazard Ratio (HR) and HR = 1.0 indicated no effect

Variables in the model	Hazard Ratio, HR (95% CI) *p*-value
Age ≥ 50 years (reference age < 50 years)	0.80 (0.55–1.15)
Sex (reference males)	*p* = 0.234
	1.06 (0.74–1.52)
Pain 6–7 (reference pain 0–5)	*p* = 0.747
	1.00 (0.77–1.99)
Pain 8–10 (reference pain 0–5)	*p* = 0.981
	1.24 (0.77–1.99)
Study Work-Up (reference MC Chiro)	*p* = 0.377
	4.65 (2.67–8.09)
Study PARA (reference MC Chiro)	*p* < 0.001
	6.17 (3.38–11.28)
	*p* < 0.001

## Discussion

We explored compliance in five studies that had used frequent SMS to collect data over a fairly long time. Overall compliance was somewhat different between the studies, ranging from 98.2% (MC Chiro) to 88.1% (PARA). However, in the great scheme of clinical research, an overall compliance of around 90% is quite remarkable. Further, a compliance around 70% at the end of a study period of 52 weeks (the lowest compliance of the Weekly studies) is in line with previous studies using SMS to collect data [7, 9], and must be considered a high figure in comparison with other means of collecting data [22, 23].

The included populations were intentionally different, in order to get a variety of factors where compliance with the SMS methodology could be explored. Four studies with working populations were included: MC Chiro, SCIP Falls, Work-Up and SPA, and two populations consisted of patients with chronic conditions, SCIP Falls and PARA. Therefore, not surprisingly, the subjects in MC Chiro, although clearly in pain when consulting for care, consisted of healthy subjects without much sick-leave. The subjects in SCIP Falls, who all lived with spinal cord injuries, reported the poorest quality of life according to the EuroQol 5 dimensions (EQ-5D) index.

Some gender differences between the populations were observed, some of which could be explained by the nature of the inclusion: PARA had the highest proportion of females, as RA is a condition affecting more females (gender ratio females/males of 80/20). In SPA, the population was also largely female, as the study was set among health care workers, traditionally female-dominated professions. In SCIP Falls, however, the participants were largely male [24]. This is also expected as the gender ratio (females/males) of SCI in Norway and Sweden is 25/75. In previous studies using the SMS technology, gender differences in compliance were minimal [6, 9].

We found that compliance was influenced to some degree by individual factors, but the factor that remained significant in the final model was “study”, i.e. the individual factors did not explain the difference in compliance as much as the study itself. We therefore need to look at the specifics of the studies to answer the questions of this paper.

Question 1: Is compliance associated with study-specific factors? There were some indications that *age and gender* did influence compliance (Fig. 3), but not as much as “study”. This “study effect” may reflect the *condition* of the subjects. Throughout, PARA experienced lower compliance than the other studies, their subjects suffering from a chronic condition, Rheumatiod Arthritis. The best compliance was found in MC Chiro, were subjects had recurrent and persistent LBP. Studying the health indicators of the subjects, participants in MC Chiro and Work-Up reported far more pain and poorer health compared to PARA participants. However, in chronic conditions such as RA, it may well be that individuals adapt to pain over the years, implying that it is not regarded as bad as if it was acute. Thus, it is difficult to conclude if *condition* may be a factor explaining compliance.

It is also possible that the *severity* of the condition explains the “study effect”, as highlighted in a previous study [9]. This was tested by using studies where categorization due to pain was possible (MC Chiro, Work-Up and PARA), but compliance was not found to be consistently different among groups of respondents with different levels in of pain intensity (Fig. 3, Tables 3, 4 and 5). Lastly, these three pain conditions are fluctuating, and it is possible that the “study effect” would be explained by *changes in the condition*. We calculated initial pain intensity development (from study start to 8 weeks) but did not find differences in compliance between those who improved, stayed the same or deteriorated (data not shown).

The “study effect” may be a result of the condition but does not seem to be attributable to severity or development of this condition, nor other available population factors. It may be due to other unmeasured differences in these populations, as we could only make comparisons across common variables.

Question 2: Is compliance associated with the SMS- methodology itself? In total 8 different questions (1 each from MC Chiro, SCIP Falls and SPA, 2 from PARA and 3 from Work-Up) were asked across the studies, all to some extent “sensitive”. However, in PARA, the SMS questions were about compliance with the intervention, i.e. physical activity. These questions were related to a socially desired behavior that was supposed to increase during the study period, while the other studies mainly measured disease-related symptoms. Failure in behavior change might certainly have been the case for lower compliance in PARA, while there is no expected failure related to the reporting of symptoms as in MC Chiro, SCIP Falls, Work-Up and SPA.

Questions have been raised concerning the influence of the technology itself on the outcome, i.e. does frequent questions about pain lead to more pain [25, 26], or is it stressful to answer frequent questions about stress [27]? However, that does not seem to be the case. In PARA, it could be proposed that the weekly SMS would act like a prompt to be more physically active, as hypothesized by others [28], but whether this prompting actually influences compliance is unknown.

Three studies were using weekly questions, one study was using questions every 2 weeks (SCIP Falls) and one (SPA) used a regimen of collecting data for 13 weeks, pausing for 12 weeks and then collecting data again for 26 weeks. If frequent questions were perceived as a burden, SCIP Falls and possibly SPA should show the highest compliance, which was not the case. It was, however, clear that holidays rendered somewhat lower compliance, as this was observed even in MC Chiro. The longest study (PARA) admittedly had the lowest compliance, but examining only the first year of PARA, compliance was lower already. Thus, the number and frequency of questions asked, as well as the study duration, did not explain differences in compliance between the studies.

In these five studies, all PI’s were given the same instructions how to oversee the data collection, send reminders and call non-compliers. It is possible that compliance with the rigorous execution of these management procedures was explaining the “study effect”, that it was a “management effect”. Indeed, early compliance was found to influence “stamina”, i.e. compliance throughout the study. As found in a previous scrutiny of the SMS method, it is important to motivate the non-responders early on [9]. In SCIP Falls, the participants were called by the investigators if answering “yes” (= I did fall), and this attention may also have contributed to the high compliance.

## Conclusions

Compliance with frequent SMS questions was high across these studies. Individual factors, number and frequency of questions or duration of the study only marginally affected compliance and only partly explained the differences in compliance between the included studies. The “study effect” found in our analysis could however be due to the nature of the variables measured (such as a socially desirable outcome) or the management of the study. Future studies should aim to follow up on non-responders early to ensure good compliance throughout, and avoid questions relating to socially desirable behavior.

## Data Availability

The datasets analysed during the current study are not publicly available due to Swedish Ethics regulations but are available from the corresponding author on reasonable request.

## Abbreviations

IT: Information Technology
SMS: Short Message System
MC: Maintenance Care
PI: Principal Investigator
LBP: Low Back Pain
SCIP: Spinal Cord Injury Prevention
SCI: Spinal Cord Injury
PARA: Physical Activity program for people with Rheumatoid Arthritis
RA: Rheumatoid Arthritis
SPA: Stress Prevention At work
VAS: Visual Analog Scale
RR: Relative Risk
CI: Confidence Interval
HR: Hazard Ratio
EQ-5D: EuroQol 5 Dimensions
